# Evaluating the Feasibility, Acceptance, and Beneficial Effects of Online Occupational Therapy for Post–COVID-19 Condition: Protocol for a Randomized Controlled Trial (ErgoLoCo Study)

**DOI:** 10.2196/50230

**Published:** 2024-05-13

**Authors:** Christina Müllenmeister, Andrea Stoelting, Dominik Schröder, Tim Schmachtenberg, Simon Ritter, Iman El-Sayed, Sandra Steffens, Frank Klawonn, Sandra Klawitter, Stefanie Homann, Marie Mikuteit, Christoph Berg, Georg Behrens, Eva Hummers, Aisha Cook, Frank Müller, Alexandra Dopfer-Jablonka, Christine Happle

**Affiliations:** 1 Department of General Practice University Medical Center Goettingen Goettingen Germany; 2 Department of Rheumatology and Immunology Hannover Medical School Hannover Germany; 3 Department of Pediatric Pulmology, Allergology and Neoantology Hannover Medical School Hannover Germany; 4 RESIST Cluster of Excellence Hannover Germany; 5 Department of Computer Science Ostfalia University of Applied Sciences Wolfenbuettel Germany; 6 Biostatistics Research Group Helmholtz Centre for Infection Research Braunschweig Germany; 7 Department of Dermatology Hannover Medical School Hannover Germany; 8 FOM University of Applied Sciences for Economics and Management Hannover Germany; 9 German Center for Infection Research, partner site Hannover-Brunswick Hannover Germany; 10 Timmcook Occupational Therapy Center Hannover Germany; 11 Department of Family Medicine Michigan State University Grand Rapids, MI United States; 12 Biomedical Research in End-stage and Obstructive Lung Disease Hannover German Center for Lung Research Hannover Germany

**Keywords:** SARS-CoV-2, COVID-19, post COVID-19 condition, pandemic, occupational therapy, cognitive deficits, online treatment, long Covid, RCT, randomized controlled trial, controlled trials, internet based, digital health, digital intervention, video, prerecorded, feasibility, acceptability, effectiveness, online therapy

## Abstract

**Background:**

Post–COVID-19 syndrome (PCS; also known as “long COVID”) is a relatively novel disease comprising physical, psychological, and cognitive complaints persisting several weeks to months after acute infection with SARS-CoV-2. Approximately 10% of patients with COVID-19 are affected by long-term symptoms. However, effective treatment strategies are lacking. The ErgoLoCo (Occupational Therapy [Ergotherapie] for Long COVID) study was designed to develop and evaluate a novel occupational therapy (OT) concept of online delivery of therapy for long COVID.

**Objective:**

The primary study objective is to assess the feasibility of the online OT intervention in PCS. Secondary aims include the evaluation of online OT concerning cognitive problems, occupational performance, and social participation.

**Methods:**

This randomized controlled interventional pilot study involves parallel mixed methods process analyses and a realist evaluation approach. A total of 80 clients with PCS aged at least 16 years will be recruited into two interventional groups. The control cohort (watch and wait) comprises 80 clients with long COVID. Treatment is provided through teletherapy (n=40) or delivery of prerecorded videos (n=40) using the same standardized OT concept twice weekly over 12 weeks. Analyses of quantitative questionnaires and qualitative interviews based on the theoretical framework of acceptability will be performed to assess feasibility. Focus group meetings will be used to assess how acceptable and helpful the intervention was to the participating occupational therapists. Standardized tests will be used to assess the initial efficacy of the intervention on neurocognitive performance; limitations in mobility, self-care, and everyday activities; pain; disabilities; quality of life (QoL); social participation; and anxiety and depression in PCS, and the possible effects of online OT on these complaints.

**Results:**

The German Ministry of Education and Research provided funding for this research in March 2022. Data collection took place from October 2022 to August 31, 2023. Data analysis will be completed by the end of April 2024. We anticipate publishing the results in the fall of 2024.

**Conclusions:**

Despite the enormous clinical need, effective and scalable treatment options for OT clients who have PCS remain scarce. The ErgoLoCo study will assess whether online-delivered OT is a feasible treatment approach in PCS. Furthermore, this study will assess the effect of the intervention on cognitive symptoms, QoL, and occupational performance and participation in everyday life. Particular emphasis will be placed on the experiences of clients and occupational therapists with digitally delivered OT. This study will pave the way for novel and effective treatment strategies in PCS.

**Trial Registration:**

German Clinical Trial Registry DRKS00029990; https://drks.de/search/de/trial/DRKS00029990

**International Registered Report Identifier (IRRID):**

PRR1-10.2196/50230

## Introduction

### Background

Post–COVID-19 syndrome (PCS; also known as post–COVID-19 condition or long COVID) is defined as chronic health challenges persisting for more than 4 weeks after an acute SARS-CoV-2 infection. PCS affects a substantial proportion of COVID-19 survivors [1], with an estimated risk of approximately 10% after each infection [2-5]. PCS is a heterogeneous condition that can include symptoms of respiratory impairment, prolonged fatigue, and neurocognitive sequelae [6-8]. Systemic, endothelial, and neuroinflammatory processes, along with reactivation of latent pathogens are suspected contributors to PCS development. However, the immunological and pathological mechanisms of PCS remain unclear and specific treatment options are scarce [9]. Importantly, due to the large number of individuals faced with this condition, PCS considerably impacts communities and health care systems worldwide [10].

Patients with PCS report substantial limitations in their everyday lives [11]. Classic PCS symptoms include problems in concentration and cognitive function, often described as “brain fog,” fatigue, and postexertional malaise. Individuals living with PCS often struggle to participate in everyday contexts such as in work, leisure, self-care, and social interaction [7,12]. Therapy guidelines such as the British National Institute for Health and Care Excellence recommend an integrative, multidisciplinary approach to treating PCS, aiming at symptom management and reduction of functional impairments [11]. Occupational therapy (OT) is central in this multidisciplinary approach [13-15], as it has been shown to improve quality of life (QoL) for individuals with PCS by enabling self-management and coping strategies and accelerating the return to relevant everyday activities in self-care, productivity, and leisure [16].

In most fields of medicine, isolation measures during the COVID-19 pandemic led to further development of online care provision [17-19]. Digital treatment strategies hold great promise for individuals with PCS, who often experience impaired mobility, as these strategies facilitate therapy access and adherence [20].

The ErgoLoCo (Occupational Therapy [Ergotherapie] for Long COVID) study will evaluate the role of digitally delivered OT treatment strategies for clients with PCS. This unblinded, randomized pilot study tests the feasibility and effect of a digitally delivered 12-week online OT program in PCS. The program is delivered either via personal digital visit sessions (teletherapy) or through prerecorded videos (video therapy), focusing on symptom management for cognitive impairment in PCS and improvement of occupational performance in relevant activities of daily life. Our purely digital and scalable approach may help bridge health care gaps and facilitate treatment access for clients with PCS.

### Objectives

This pilot study hypothesizes that an online-administered OT treatment program for individuals affected by PCS is a suitable form of intervention to reduce symptoms and improve occupational performance and QoL. The primary objective is to assess the feasibility of the ErgoLoCo concept by collecting data on participation rates, participant satisfaction, and experience. Secondary aims include analyzing changes in cognitive performance, occupational performance, QoL, and social participation. We here outline the study design and protocol according to the CONSORT (Consolidated Statement of Reporting Trials) guidelines [21].

## Methods

### Study Setting

The ErgoLoCo study centers are located in Lower Saxony, Germany, at the Department for Rheumatology and Immunology of Hannover Medical School and the Department of General Practice of the University Medical Center Goettingen. Data analyses will be conducted at the Ostfalia University of Applied Sciences in Braunschweig. Lower Saxony is Germany’s second-largest federal state with approximately 8 million residents [22]. The number of people affected by PCS in this federal state is unclear. Based on data from a study on cases of illness and death associated with COVID-19 in Lower Saxony, approximately 3,528,782 people had experienced a reported infection with SARS-CoV-2 by the end of 2022 [23].

### Study Design

This pilot study is a 12-week unblinded randomized controlled trial with two intervention groups (group 1: teletherapy/live online; group 2: video on demand) in comparison to a control group (group 3: watch and wait). The study is designed to evaluate feasibility and acceptability, as well as to obtain preliminary evidence on efficacy. The observational period comprises 24 weeks: a baseline assessment (T0) and a 12-week intervention phase with subsequent online questionnaire and testing (T1). At week 24 (T2), study participants will be asked to fill out questionnaires and participate in interviews, and a second round of online testing will be conducted. Given the digital nature of ErgoLoCo, the entire study process, including enrollment, treatment, interviews, and testing, will be performed through online visits.

### Participants

The study will enroll individuals 16 years and older with PCS symptoms persisting at least 4 weeks after confirmed SARS-CoV-2 infection. We will enroll a convenience sample of 120-150 participants.

### Eligibility Criteria

Inclusion criteria are as follows: (1) 16 years or older, (2) affected by persisting health problems beyond 4 weeks after SARS-CoV-2 infection (confirmed either by polymerase chain reaction or rapid antigen testing), (3) personal reporting of subjective cognitive symptoms such as brain fog and fatigue ≥5/10 on a Likert scale, (4) having access to an appropriate technical device to participate in the intervention (eg, PC, tablet, or smartphone with internet access), and (5) provision of written informed consent to participate in the study (in clients <18 years of age, additional written consent is to be provided by legal guardians). Individuals not meeting the above-mentioned inclusion criteria and those lacking the willingness or ability to complete the program consisting of either teletherapy or video sessions will be excluded from the study.

### Recruitment

Potential participants will be recruited via the homepage of participating hospitals and research departments and a prior observational study on PCS (DEFEAT Corona study) [24,25]. Moreover, local private practices and clinics throughout Northern Germany and beyond will be asked to share information on the study with affected clients in their care. Clients interested in participating in the study are asked to register on the DEFEAT study webpage [25].

### Participant Screening

Study eligibility will be evaluated by online questionnaires on self-perceived PCS-related cognitive impairment (eg, brain fog and fatigue scores ≥5/10 or higher on a Likert scale). Eligible participants will receive comprehensive study information as well as an invitation to book an online appointment by email. Upon the first digital visit, study staff will provide further information, discuss possible questions, and recheck eligibility criteria. After obtaining written informed consent by digital signature on an online study inclusion form (including signature of a legal guardian for participants below the age of 18 years), data on age, gender, and health status are collected. Furthermore, information on social participation and subjective well-being are obtained. Participants are also tested for cognitive functions and impairments in daily life (Table 1).

**Table 1:** Schedule for enrollment and assessment.

Stage and outcome		Instrument	Time point
Eligibility screen		Online questionnaire	Enrollment
Informed consent		Digital visit	Enrollment
Randomization		Digital visit	Enrollment
**Mixed methods study on acceptability and feasibility (primary outcome)**			
	Acceptance and feasibility of the online program	Semistructured interviews with 12 participants of each treatment group	Week 6 and week 12
	Acceptance and feasibility of the online program	Questionnaire assessing 35 Likert-scaled items developed based on the theoretical framework of acceptability in all participants [26]	Week 24
	Acceptance and feasibility of the online program	Focus group meeting notes on acceptance and feasibility of the online program by occupational therapists providing live-online sessions	Week 12
**Neurocognitive and occupational impairments (secondary outcomes)**			
	Self-perception of occupational performance in everyday life	Canadian Occupational Performance Measure [27]	Enrollment and week 12
	Memory and cognitive impairment	Wilde-Intelligence test-2, modules MF^a^ and ER^b^ [28]	Enrollment, week 12, and week 24
	Impairment in daily life activities, family and social life, stress, and sexual activity	Index for the Assessment of Health Impairments [29]	Enrollment, week 12, and week 24
	Physical, mental, and social effects of neurological conditions	Quality of Life in Neurological Disorders [30]	Enrollment, week 12, and week 24
	Deficits in concentration, attention [31], and mental speed	D2 test of attention-revised [31]	Enrollment, week 12, and week 24
	Mobility, self-care, usual activities, pain, disabilities, anxiety, and depression	EQ-5D-5L [32]	Enrollment, week 12, and week 24
**General and health information**			
	Date of enrollment, demographic data (eg, age, sex, education level, residential and housing status, care level, disabilities, migration status)	Online questionnaire	Enrollment
	Preexisting health conditions, medication, smoking, SARS-CoV-2 infections	Online questionnaire	Enrollment
	Vaccination status (time point and type of COVID-19 vaccinations)	Online questionnaire	Enrollment

^a^MF: memory function.

^b^ER: embedded math exercises.

### Randomization

**Figure 1:**
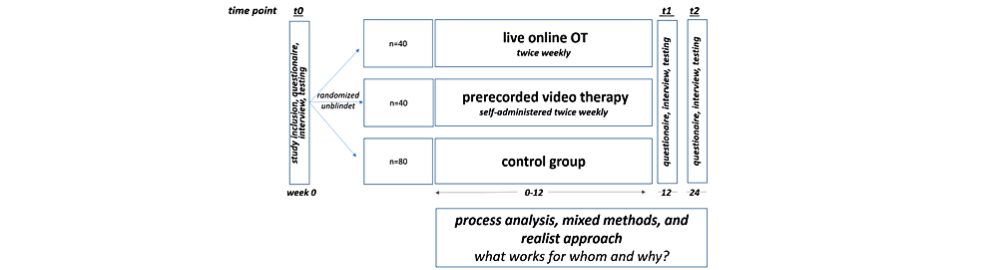
Process flowchart of the ErgoLoCo study. OT: occupational therapy.

Directly after enrollment, urn randomization will be carried out to ensure a balanced distribution of the participants across groups, as shown in Figure 1. Randomization will be performed by an independent study group member not involved in the study design or OT delivery.

### Intervention

The OT concept was designed using the Medical Research Council Framework, ensuring evidence-based and theory-based OT-specific approaches [33]. These approaches will be identified using a literature review, including the English, Dutch, and German literature. Furthermore, we will include stakeholder experiences in treating clients with PCS in inpatient and outpatient settings in Germany, Austria, and Switzerland. The OT concept will include 24 30-minute standardized therapy sessions. Each session will be delivered twice a week over 12 weeks. Therapy sessions consist of OT-specific instructions for managing PCS symptoms, which will be tailored to individual aims defined for each OT recipient upon entering and throughout the program. Participants will receive an accompanying workbook with materials to support therapy transfer into everyday life.

### Intervention Delivery

After randomization, each participant is interviewed by an occupational therapist to assess occupational performance challenges in their everyday occupations, using the German version of the Canadian Occupational Performance Measure (COPM) [27]. The COPM has been validated in various OT intervention studies, which provides metrics on changes in occupational performance, satisfaction and importance, and enhancing transparency in OT outcomes [26,34-37].

The teletherapy/live online group (group 1; n=40) will undergo online OT by a professional occupational therapist via teletherapy twice week using the medical communication and conference software medflex, which ensures data security compliant with the German General Data Protection Guidelines, Datenschutz-Grundverordnung. Video group participants (group 2; n=40) will receive digital OT through prerecorded videos twice weekly. Group 2 participants will receive emails every 2 weeks with links to four online videos illustrating the next intervention module. The participants in this group are asked to watch two videos per week. Intervention groups 1 and 2 will receive both a printed and digital version of a workbook that visualizes and supports all tasks performed throughout the program. The control group (group 3; n=80) is a watch-and-wait group, and these participants will receive the video-on-demand- materials after study completion.

### Outcomes

#### Primary Study End Points: Feasibility and Acceptability

To assess feasibility and acceptability, we will use a realist evaluation approach [38]. Realist evaluation emphasizes the interplay of contexts through social interactions that engage with the intervention. In alignment with this method, this study aims to assess the effects and influences of the digitally provided OT program on the health status of the study participants and the feasibility and acceptance of both digital approaches from the user’s perspective. Therefore, we will collect quantitative data regarding participant rates in the follow-up examinations, participant surveys, and qualitative in-depth interviews about the participants’ participation experiences.

All participants in both intervention groups (n=80) will be asked via email to complete an online questionnaire with four closed (Likert-scaled) questions and two open questions on handling the digital intervention after each of the six intervention modules. This approach will enable the individual evaluation of different program parts. Questions include: “Were you able to apply the content of the last module in your everyday life?” “What was particularly beneficial for you in the last module?” and “How satisfied were you with the last module?”.

Moreover, all participants will be asked to complete an online questionnaire including 35 Likert-scaled items developed within discursive circles by research team members based on the theoretical framework of acceptability (TFA) [39,40]. The TFA proposes a theoretical framework for assessing the acceptability of health interventions and consists of seven component constructs: affective attitude, burden, ethicality, intervention coherence, opportunity costs, perceived effectiveness, and self-efficacy [40].

To enrich quantitative data with the first-hand experiences of the study participants, a subsample of at least 12 study participants of intervention groups 1 and 2 will be invited to share their personal experiences regarding the acceptability and feasibility of the program at week 6 and week 12. These semistructured interviews will last approximately 45 to 60 minutes.

To capture the occupational therapists’ experiences with OT teletherapy, an online focus group interview will be conducted with all occupational therapists working in the study (N=8). A moderation guide will be developed to help facilitate the engagement of participants in a discussion on the feasibility of the treatment concept, acceptance, and recommendations for further intervention development. All conducted interviews, as well as the audio-recorded focus group meeting, will be transcribed according to the simplified rules of Dresing and Pehl [41] and will be analyzed using thematic content analysis [42] by a team of three researchers.

#### Secondary End Points

Secondary outcomes will involve the analysis of possible efficacy outcomes such as cognitive performance, health-related QoL, social participation, occupational performance and satisfaction in daily life, and memory and concentration abilities. All measurements used for data collection and the time points at which they will be collected are depicted in Table 1.

### Analytical Approach

#### Power and Sample Size

The main focus of this study is to assess the feasibility and acceptability of the intervention. Therefore, efficacy analyses with regard to secondary outcomes will only be exploratory. The pilot study is not sufficiently powered to evaluate the effectiveness of the intervention. However, exploratory analyses will address potential differences in outcomes of cognitive function, QoL, occupational performance, and social participation between the intervention and control groups. No previous reports on OT concepts in PCS or online OT in general were available that could have been used to estimate sample sizes. As current practice standards suggest that formal sample size calculations are unsuitable for feasibility trials, the convenience sample of 120-150 participants was selected pragmatically [43].

#### Statistical Analysis Plan

All quantitative data will be prepared using Microsoft Excel or SPSS software and analyzed with R statistical packages. Data visualizations will be created using GraphPad Prism and R software tools. Analysis of the following items is planned from the time points 0 weeks (T0), 12 weeks (T1), and 24 weeks (T2): impairments in daily life activities, family and social life, stress, and sexual activity, and deficits in concentration, attention, and mental speed, particularly focusing on visual attention, physical, mental, and social symptoms of PCS, including concentration, attention, mental speed, mobility, self-care, usual activities, pain, disabilities, anxiety, and depression. An intention-to-treat approach will be used. Estimates and 95% CIs will be computed for the mean, median, and selected lower quantiles. To compare time points, *t*, Wilcoxon-Mann-Whitney, and Kolmogorov-Smirnov tests will be applied, including corrections for multiple testing. Scores derived from questionnaires will be analyzed concerning change over time using *t* and Wilcoxon-Mann-Whitney tests, including corrections for multiple testing. Effect sizes will also be computed. Furthermore, at T0, T1, and T2, we will assess whether certain factors such as age or education influence the feasibility of therapy experiences using *t*, Wilcoxon-Mann-Whitney, and Fisher exact tests as appropriate.

#### Analysis of Qualitative Data

All interviews and the focus group will be audio-recorded, pseudonymized, transcribed verbatim, and analyzed using thematic content analysis following the approach of Kuckartz and Rädiker [42], assisted by the MAXQDA analysis software.

### Data Management

All assessment measures will be completed online. The data collected will be categorized in four data sets: (1) personal data such as name and email address; (2) sociodemographic data (eg, clinical characterization, age, gender, migration background, health status, and symptoms), as well as attitudes toward vaccination and experiences with COVID-19 vaccination; (3) test results (scores in cognitive assessments and occupational performance assessments); and (4) qualitative data such as interview transcripts and written interview documentation. Data from the first 3 categories will be incorporated into two independent databases, which are stored separately for data protection reasons.

### Ethical Considerations

The study is registered at the German Clinical Trial Register (DRKS00029990), an approved primary study registry within the World Health Organization network, and has been reviewed by the institutional review boards of participating sites (eg, Hannover Medical School; DEFEAT Corona No. 9948_BO_K_2021). The study is designed as an interventional online pilot study. Interested participants will obtain information about participation in advance on the internet and will be fully informed personally by a study team member about possible benefits and risks of study participation before inclusion. Written informed consent will be obtained from all study participants (as well as from their legal guardians for those younger than 18 years). Additionally, each participant will receive detailed written information on the study and data management procedures. All participants will be informed that their study participation is entirely voluntary and that they can withdraw participation consent at any time without disclosing reasons. Contact information to reach out to the study team for questions via email, telephone, or online meetings at any point during the study will be provided upon enrollment. All data will be collected in pseudonymized form and designated data safety measures have been developed to ensure the privacy of each participant. Personal data will be separated from all other collected data and stored in encrypted form. Qualitative data will be exclusively processed at the University Medical Center Göttingen, and other project team members cannot access these data. Following the transcription of interview notes, transcripts will be pseudonymized and any information (such as location and names of participants) that could enable retrograde identification will be eliminated (ensuring factual anonymization). Audio raw data/video data will be subsequently destroyed. Transcripts will be stored on digital media with password protection in a lockable file cabinet. If a participant does not consent to the use of factually anonymized data for further research projects, transcripts will be destroyed after 10 years. Study participation can be realized entirely from home without any physical or infection risk.

## Results

In this study, we aim to evaluate the feasibility of digital OT as a treatment for clients with PCS. Exploratory analyses will address possible cognitive and performance improvements, QoL, and social participation. The German Ministry of Education and Research provided funding for this research in March 2022 (FKZ 01EP2103A). Data collection is scheduled to take place from October 2022 to August 2023, followed by data analysis between September 2023 and April 2024. We anticipate publishing the results in the fall of 2024.

## Discussion

The ErgoLoCo study is a randomized controlled interventional pilot study designed to gain insight into new therapy options for people affected by long COVID. The intervention was developed to improve occupational performance in relevant everyday occupations and the self-management of PCS-related challenges. As a pilot study, ErgoLoCo is not powered to show treatment efficacy. However, it will provide the first insights into the acceptance and feasibility of a novel OT intervention. The mixed methods approach will provide detailed information on which parts of the intervention will likely impact the well-being of clients with PCS.

Although the current study focuses on long COVID, other medical conditions with similar symptoms, such as myalgic encephalomyelitis or chronic fatigue syndrome, may also benefit from our approach in the future.

A short description of the ErgoLoCo study design will be included in each publication by the study group and this protocol will be referenced. If changes in the study design are necessary, these will be brought to the attention of the institutional review board and will be amended to the study protocol.

The ErgoLoCo pilot study will contribute to developing treatment strategies in PCS, an emerging disease with increasing prevalence, strong socioeconomic impact, and high clinical need.

## Abbreviations

CONSORT: Consolidated Statement of Reporting Trials
COPM: Canadian Occupational Performance Measure
ErgoLoCo: Occupational Therapy (Ergotherapie) for Long COVID
OT: occupational therapy
PCS: post–COVID-19 syndrome
QoL: quality of life
TFA: theoretical framework of acceptability

## Data Availability

The results of this study will be published, preferably in open access journals. Further data and information can be obtained by the study team upon reasonable request.
